# A Quantitative Trait Nucleotide-Based Genomic Selection Strategy for Seed Oil and Protein Content in Soybean

**DOI:** 10.3390/plants15091296

**Published:** 2026-04-22

**Authors:** Guang Li, Huangkai Zhou, Javaid Akhter Bhat, Kuanqiang Tang, Jiantian Leng, Xianzhong Feng, Xiangfeng Wang, Suxin Yang

**Affiliations:** 1 Key Laboratory of Soybean Molecular Design Breeding, Northeast Institute of Geography and Agroecology, Chinese Academy of Sciences, Changchun 130102, China; 2 Jilin Academy of Agricultural Sciences (China Agricultural Science and Technology Northeast Innovation Center), Soybean Research Institute, Changchun 130033, China; 3 National Maize Improvement Center, Department of Crop Genomics and Bioinformatics, College of Agronomy and Biotechnology, China Agricultural University, Beijing 100193, China

**Keywords:** soybean, genomic selection, quantitative trait nucleotides, seed oil, seed protein

## Abstract

In recent years, genomic selection (GS) has been widely adopted in plant breeding; however, its practical application is constrained by the high cost of genotyping large segregating populations. To address this issue, this study employed a Quantitative Trait Nucleotide (QTN)-assisted GS strategy to evaluate its efficiency in reducing genotyping costs for soybean seed oil content (OC) and protein content (PC). Based on six multi-parent F_4_ populations (*n* = 4404) derived from seven elite soybean cultivars, which were genotyped using a 20K SNP chip, we identified 83 and 110 QTNs that were significantly associated with OC and PC, respectively. Among these loci, 37 and 62 QTNs were specific to OC and PC, respectively. Genomic prediction accuracies were evaluated across different training population (TP) sizes using three marker panels: genome-wide SNPs, all detected QTNs, and trait-specific QTNs. The panel consisting of all detected QTNs exhibited significantly higher prediction accuracy than the other two panels, except for PC when using 90% of the population as the training set. Phenotypic verification of the selected individuals showed that the PC-specific QTN panel yielded higher PC values and increased OC + PC values compared with the other marker panels. These results demonstrate that a small set of QTNs provides a cost-effective approach for genomic selection in practical soybean breeding programs.

## 1. Introduction

Genomic selection (GS) is an effective breeding improvement method for complex quantitative traits in plants controlled by multiple genes [1,2]. Different GS models, including direct methods, such as genomic best linear unbiased prediction (GBLUP) [3], single-step best linear unbiased prediction (ssBLUP) [4,5], standard Best Linear Unbiased Prediction (sBLUP), and combined Best Linear Unbiased Prediction (cBLUP) [6], and indirect methods, such as ridge regression best linear unbiased prediction (rrBLUP) [7], BayesA, BayesB, BayesCπ, BayesDπ [8], and Bayesian LASSO [9], have been developed. Previous studies have demonstrated that distinct models yield divergent prediction values for various traits, primarily owing to differences in the assumptions underlying marker effect distributions, which in turn influence the total variance [1,2,10].

While numerous models are available for GS, its commercial application in plant breeding is hindered by a critical challenge i.e., high genotyping costs. Population size and marker density serve as key factors influencing both cost efficiency and prediction accuracy [11,12,13]. Research has demonstrated that in aquaculture species, single-nucleotide polymorphism (SNP) panels comprising 1000–2000 markers yield selection accuracies comparable to those attained via high-density genotyping [11]. An increasing number of studies have recently focused on employing low-density marker panels combined with small training populations (TPs) to reduce genotyping costs for GS in crops [14,15,16,17].

Soybean (*Glycine max* L.) is a major cash crop cultivated worldwide, primarily owing to its seeds being abundant in edible protein and oil. On average, soybean seeds contain approximately 40% protein content (PC) and 20% oil content (OC) [18]. The OC and PC traits in soybean are well characterized and exhibit a significant negative correlation, with the relevant functional genes exerting opposing regulatory effects on them [19,20,21]. For such antagonistic traits, the artificial selection for one trait invariably leads to the reduction in the other [22]. Both OC and PC are complex quantitative traits in soybean controlled by multiple genes [23,24]. Whole-genome marker association analysis has been effectively applied to identify QTLs and functional genes underlying seed PC and OC traits in soybean [25,26,27,28,29,30]. Although hundreds of QTLs associated with OC and PC have been documented in SoyBase (http://www.soybase.org), the genetic mechanisms underlying these traits remain to be fully elucidated. Thus, high-resolution dissection of the detailed genetic architecture of OC and PC will facilitate the development of strategies to mitigate their negative correlation.

In this study, our main objectives were to develop a strategy for reducing the genotyping cost of GS, thereby promoting its application in commercial breeding. To this end, we used a GS population consisting of 4404 individual F_4_ plants and selected two negatively correlated traits, OC and PC, to demonstrate the effectiveness of our strategy.

## 2. Results

### 2.1. Population Structure Analysis of the Genomic Selection Population

**Figure 1:**
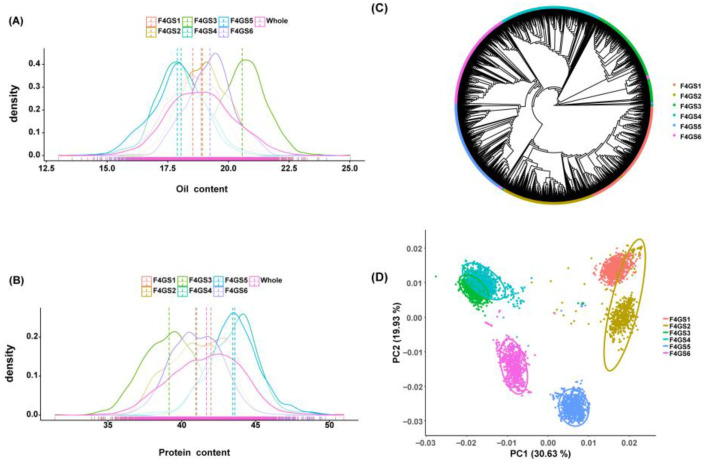
Oil content (**A**) and protein content (**B**) distribution of six populations. The *X*-axis represents the oil (%) or protein content (%); density curves with different colors represent the distribution of the oil or protein content in different populations; vertical dashed lines represent the average trait values for different populations. Phylogenetic analysis (**C**) and principal component analysis (PCA) of six populations are shown (**D**). In the PCA scatterplot, different colors represent individuals from different populations, and the ellipses represent the standard error ranges of PCA1 and PCA2 for each population.

A total of 4404 soybean genotypes were used in this study, which were derived from six F_4_ single-hybrid populations (Table S1). These six populations, designated as F_4_GS1-6, were developed from six bi-parental crosses involving three male parents and four female parents (Table S1). Semi-sib relationships were prevalent among these populations, and the agronomic performance of quality-related traits of these parents is presented in Table S1. Two key traits, namely OC and PC, exhibited an approximately normal distribution across all six F_4_ segregating populations, as well as the combined population (Figure 1 and Figure S2, and Table S1). With respect to OC, the F_4_GS3 population had the highest average value (20.56%), while the F_4_GS4 population had the lowest (18.05%). For PC, the F_4_GS5 population displayed the highest average content (43.58%), and the F_4_GS3 population had the lowest (39.15%). OC and PC showed a significant negative correlation in each population, with Pearson’s correlation coefficients ranging from −0.76 to −0.86 (Table S1).

To address inter-population cross-prediction challenges, we pooled the six F_4_ populations for downstream marker-trait association (MTA) and GS analyses. Phylogenetic analysis and principal component analysis (PCA) revealed that individuals from the same hybrid clustered together, while F_4_ populations sharing a common parent and half-sib families (e.g., “F_4_GS1 and F_4_GS2”, “F_4_GS3 and F_4_GS4”) grouped closely (Figure 1C,D). PCA and phylogenetic results were consistent (Figure 1D), confirming the six F_4_ populations were genetically distinct but related. This structure characterized by distinct but related semi-sib subpopulations was incorporated into MTA and GS. It minimized false MTAs and facilitated the evaluation of GS model transferability, forming a feedback loop.

### 2.2. QTN Identification for OC and PC via Whole-Genome Marker Association Analysis

**Figure 2:**
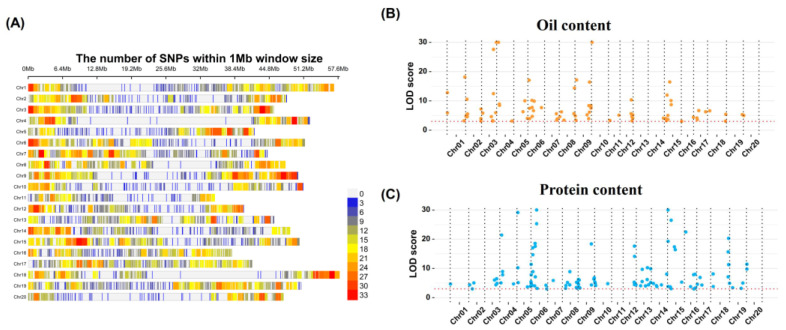
Position distribution of 9942 SNP markers in the soybean genome, from the customized panel (**A**). Distribution of quantitative trait nucleotides (QTNs) of the two traits, namely OC (**B**) and PC (**C**), identified via MTA analysis across the soybean genome. The *X*-axis represents chromosomal coordinates, and the *Y*-axis represents the significance level of QTNs. In panel (**B**), yellow dots represent QTNs associated with oil content (OC); in panel (**C**), blue dots represent QTNs associated with protein content (PC).

We genotyped the soybean population using a customized soybean genotyping panel, which included 20,659 single-nucleotide polymorphism (SNP) markers mainly derived from the genic/protein-coding regions of the soybean genome. After quality control filtering, we retained 9942 high-quality SNPs for further genetic analysis. For these 9942 SNPs, we estimated marker density as the number of SNPs per 1 Mb contiguous window in the soybean genome. Our results showed that these SNP markers almost covered the entire soybean genome, with relatively lower density near centromeres (Figure 2A).

To reduce the number of markers for GS, we performed MTA analysis on a combined population of 4404 lines from six individual populations, aiming to detect quantitative trait nucleotides (QTNs) associated with OC and PC (Table S1). Six multi-locus association models (mrMLM, FASTmrMLM, FASTmrEMMA, ISIS EM-BLASSO, pLARmEB, and pKWmEB) were used to enhance detection power and result reliability. Among the six models, OC-associated QTNs detected ranged from 11 to 41; and 83 non-redundant OC QTNs were retained (detected by ≥2 methods, or single-method LOD ≥ 3.0), that are distributed across all soybean chromosomes except chr.13 (Figure 2B; Table S2). For PC, QTNs detected ranged from 10 to 49; and 110 non-redundant PC QTNs were retained (detected by ≥2 methods, or single-method LOD ≥ 3.0), that are present on all chromosomes except chr.11 (Figure 2C; Table S2).

Comparison of the chromosomal positions of QTNs demonstrated that 46 out of 83 OC QTNs and 48 out of 110 PC QTNs were located within 25 shared genomic regions (genomic distance < 1 Mb), while 37 OC QTNs and 62 PC QTNs were identified as trait-specific loci (Table S2). The overlapping proportions, accounting for 55.40% of OC QTNs and 43.60% of PC QTNs, thus revealing the genetic basis underlying the antagonistic relationship between these two traits.

### 2.3. Effects of Model Selection on GS Prediction Accuracy

**Figure 3:**
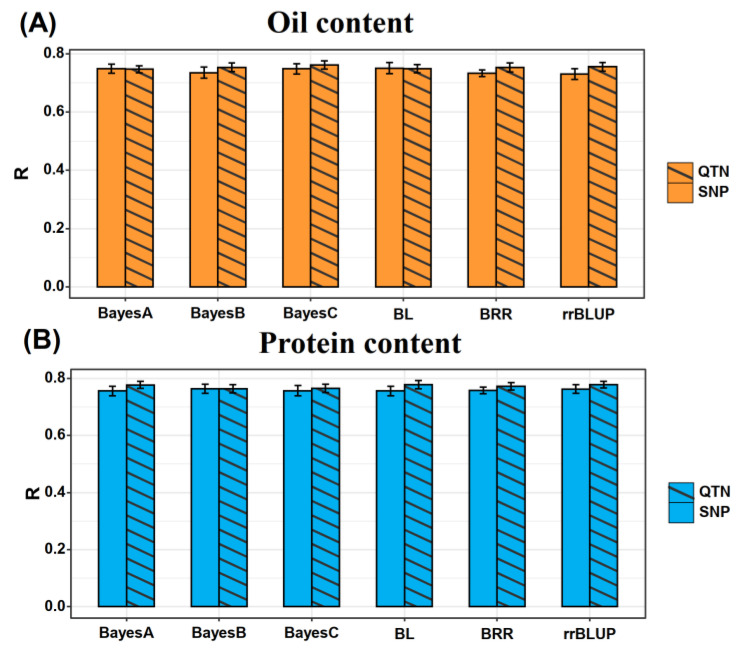
GS prediction based on genome-wide SNP markers and QTNs for OC (**A**) and PC (**B**) using six methods. The height of the histogram represents the GS prediction accuracy, and the error bar represents the standard error of the prediction accuracy.

The prediction performance of six GS models, namely Bayes A, Bayes B, Bayes C, Bayesian Ridge Regression (BRR), Bayesian Lasso (BL), and rrBLUP, were evaluated using 10 repetitions of cross-validation. A total of 90% of the population (*n* = 3964) was assigned as the test set, with the correlation between predicted and observed phenotypic values serving as the key evaluation metric (Figure 3 and Figure S3).

For the OC trait, the average prediction accuracies achieved by the six models using all SNP markers were 0.749, 0.735, 0.748, 0.750, 0.733, and 0.731, respectively. In contrast, when the 83 OC-associated QTNs were employed instead of all SNP markers, the corresponding average prediction accuracies were 0.747, 0.753, 0.761, 0.749, 0.753, and 0.755, respectively (Figure 3A).

A similar trend was observed for the PC trait. When all SNP markers were used, the average prediction accuracies of the six models were 0.756, 0.764, 0.756, 0.756, 0.757, and 0.763, respectively. In comparison, the use of 110 PC-related QTNs resulted in average prediction accuracies of 0.777, 0.764, 0.765, 0.778, 0.772, and 0.778 (Figure 3B).

Furthermore, paired *t*-tests with Bonferroni correction revealed no significant differences in prediction performance among the six models, regardless of all SNPs or trait-associated QTNs used (Table S3). Notably, while the identified QTN sets included several variants with low PVE (<0.01%)—which could represent minor QTLs or statistical noise—their exclusion (2 for OC and 4 for PC) had negligible impacts on GS accuracy (change < 0.02, *p >* 0.01; Table S4). Robustness analysis (Figure S4) further revealed nearly identical CV% profiles between the full and filtered QTN sets across all TP ratios. This indicates that these minor-effect variants do not introduce detrimental noise or compromise model stability. Consequently, the full QTN sets were retained for all subsequent analyses to ensure a comprehensive evaluation of the genetic architecture.

### 2.4. Effects of Training Population Size and Genotyping Marker Types on Prediction Accuracy

**Figure 4:**
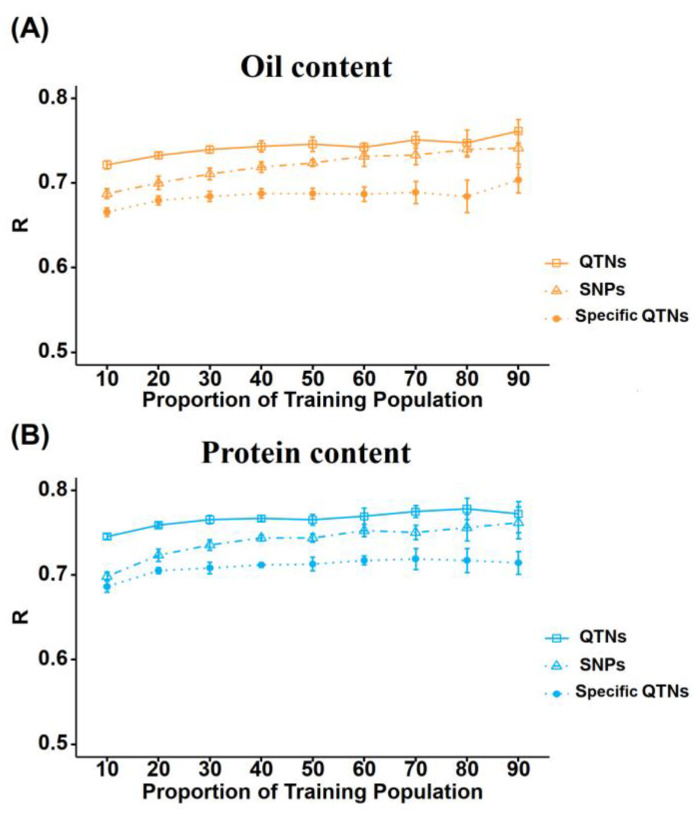
Prediction accuracy of OC (**A**) and PC (**B**) with different training population sizes (accounting for 10–90% of the whole population) and comparison of prediction accuracy of genomic selection of oil content and protein content (using trait-specific QTNs, all QTNs and all SNPs).

Training population (TP) size and genotyping marker quantity are critical factors influencing the cost of GS. We applied the rrBLUP model to assess their impacts on the predictive accuracy of OC and PC, evaluating three marker panels: 9942 SNPs (GSall), 83 OC-associated and 110 PC-associated QTNs (GSQTN), together with 37 OC-specific and 62 PC-specific QTNs (Specific QTNs) (Figure 4; Table S5).

When the TP proportion rose from 10% to 90% (test plants from 440 to 3964), all marker sets presented elevated predictive accuracy. GSall boosted OC accuracy by 0.054 and PC by 0.064, while GSQTN improved OC and PC accuracy by 0.040 and 0.026, respectively (Figure 4A). The smaller accuracy increment of GSQTN with expanding TP confirmed that QTN markers can realize favorable predictive performance with a limited TP size. In contrast, specific QTNs showed overall low accuracy, with mean values ranging from 0.686 to 0.717 for both traits, and its predictive performance remained stable with negligible responses to TP size changes (Figure 4A,B).

According to Tukey’s HSD test (*p <* 0.05), GSQTN exhibited significantly higher accuracy than GSall for both traits at small TP sizes (10–30%), and this advantage faded as TP increased (Table S5). At TP = 90%, GSall and GSQTN performed similarly for PC, yet GSQTN still maintained significantly superior accuracy for OC. Specific QTNs fell into an independent statistical group in post hoc tests, with accuracy markedly lower than the other two panels. Nonetheless, its stable predictivity facilitates the effective capture of core genetic signals (Figure 4).

### 2.5. Verification of Trait-Associated and Trait-Specific QTNs in Genomic Selection

**Figure 5:**
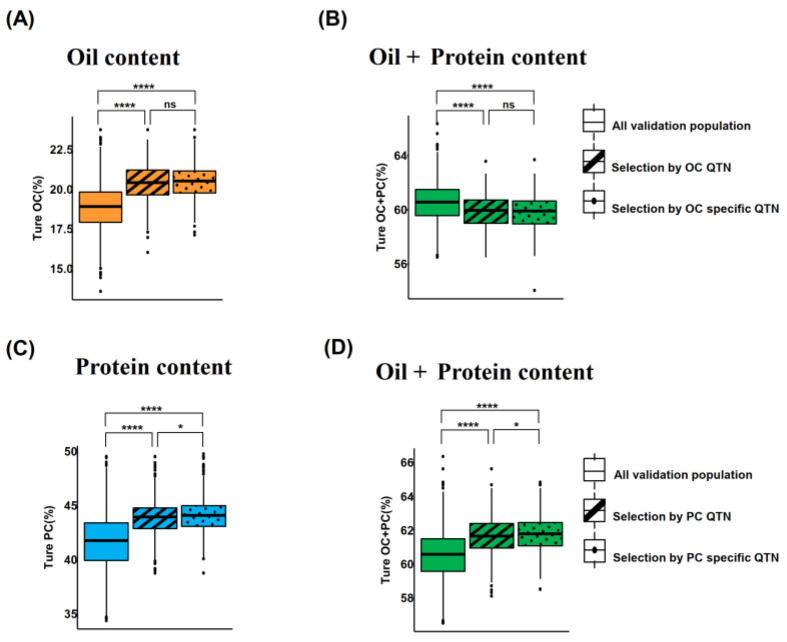
Effects of subgroup screening using the GS model with different QTN sets. (**A**,**B**) True OC and PC distributions for the subgroups of the top 20% of individuals selected based on genomic selection using the two OC QTN sets. (**C**,**D**) True PC and OC distributions for the subgroups of the top 20% of individuals selected based on genomic selection using the two PC QTN sets. Comparison among groups in the figure was performed using a *t*-test; **** *p* < 0.0001, * *p* < 0.05, and ns represents no significant difference.

To verify the effectiveness of trait-associated QTNs and trait-specific QTNs in GS applications, half of the F_4_ population was assigned as the TP (*n* = 2202) using the rrBLUP model, while the other half served as the validation population (VP) to evaluate the performance differences between these two marker sets (Figure 5).

For the OC trait, 37 OC-specific QTNs and 83 OC-associated QTNs were used to compare the predicted OC values of the top 20% individuals (*n* = 440) with higher OC in the VP against their actual measured OC values. The results showed that both subgroups exhibited significantly higher OC levels than the average OC of the entire VP, confirming the effectiveness of GS using QTNs for oil trait selection (Figure 5A). No significant difference in OC was observed between the two subgroups, indicating that 83 OC-associated QTNs did not improve the prediction accuracy. Additionally, both subgroups displayed lower OC + PC values compared to the VP average (Figure 5B), reflecting a negative genetic correlation between OC and PC. This finding suggested that the limited number of OC-specific QTNs was insufficient to counteract the antagonistic effects of common QTNs.

For the PC trait, 62 PC-specific QTNs and 110 PC-associated QTNs were employed. Both selection panels successfully identified VP subgroups with significantly increased PC levels (Figure 5C). The subgroup selected based on PC-specific QTNs had an average PC of 44.10%, which was higher than the 43.80% observed in the subgroup selected using all PC-associated QTNs (*p* = 0.018). This result indicates that common QTNs attenuated the predictive signal of PC

The OC + PC value of the PC-specific QTN subgroup reached 61.80%, which was significantly higher than the 61.60% of the all-QTN subgroup (*p* = 0.013). This outcome is attributed to the greater increase in PC, thereby optimizing seed quality (Figure 5D). These findings demonstrate that PC trait-specific QTNs can alleviate the OC-PC trade-off, with a more prominent effect on OC + PC than OC trait-specific QTNs (Figure 5B,D).

## 3. Discussion

Cost-effective GS strategies are essential for the extensive implementation of GS in plant breeding programs [31,32], with their value assessed by the genetic gain attained per unit cost [33]. Population-specific linkage disequilibrium (LD) patterns enable the development of cost-efficient genomic prediction models employing low-density SNP panels [34]. The optimization of the TP is designed to achieve a balance between maximizing genetic gain via elevated genomic prediction precision and cutting phenotyping costs by shrinking the TP scale [35]. This study proposes an innovative approach: detecting QTNs via MTA in a moderately sized TP, and subsequently establishing low-density marker panels for high-throughput screening in practical GS applications.

### 3.1. Targeted QTN Sets as an Alternative to Dense Genome-Wide Markers for GS Application

A key advantage of QTN-based GS relative to genome-wide marker-based strategies resides in its maintenance of model simplicity and cost-effectiveness. Our findings revealed that six conventional GS models (including rrBLUP, BayesA, BayesB, BRR, BL and BayesC) exhibited comparable predictive performance for OC and PC, irrespective of whether they were trained using 9942 genome-wide markers (GSall) or 83–110 QTNs (GSQTN). Strikingly, GSQTN surpassed GSall in predictive accuracy when small TPs were utilized: with a 10% TP, GSQTN attained an OC prediction accuracy of 0.721, exceeding that of GSall implemented with larger training populations (Figure 4). This superiority can be ascribed to the enrichment of genetic signals derived from stable, trait-specific QTNs and the concomitant reduction in background noise [36,37,38]. Although the overall prediction accuracy (PA) of GSQTN was moderate, the stable and trait-targeted characteristics of specific QTN panels equip them with an enhanced ability to capture the core genetic determinants of target traits.

### 3.2. Trait-Specific QTNs in Mitigating OC-PC Trade-Off

Soybean OC and PC exhibit a strong negative correlation, which constitutes a major bottleneck in soybean breeding [22,39]. This negative correlation has traditionally been ascribed to pleiotropy and tight genetic linkage between loci regulating the two traits [40]. Similar trade-offs are prevalent in wheat, where GS indices have been employed to decouple grain yield from protein quality. By integrating dough rheological traits into GS models [41] or using genomic-assisted approaches to optimize nitrogen utilization [42], breeders can identify genotypes that balance yield and protein more effectively than classical phenotypic selection. These advancements underscore the potential of genomic strategies in mitigating negative correlations between complex traits. Trait-specific QTNs offer an alternative approach to breaking unfavorable linkages without relying on large segregating populations: PC-specific QTNs achieved a PC of 44.10% (vs. 43.80% with all PC QTNs, *p* = 0.018) with a comparable level of OC loss, and a higher combined OC + PC (61.80% vs. 61.60%, *p* = 0.013) (Figure 5D), thereby enhancing seed value by effectively balancing the inherent OC-PC trade-off.

## 4. Materials and Methods

A schematic workflow diagram (Figure S1) outlines the experimental design and analytical pipeline, including population development, phenotypic/genotypic data collection, MTA, GS model comparison, TP optimization, and QTN effect evaluation on the OC–PC trade-off.

### 4.1. Plant Materials

Seven soybean cultivars from Northeast China were used to construct six F_4_ populations (Table S1). Their PCs/OCs were: FNGS0852 (44.64%/19.35%), FNGS0225 (40.95%/21.60%), FNGS0239 (43.46%/19.67%), FNGS0217 (40.72%/22.01%), FNGS0256 (44.35%/20.94%), FNGS0280 (44.84%/18.52%), and FNGS0301 (46.69%/17.90%). Populations and parents were planted in Jiamusi (46°48′ N, 130°22′ E) in 2019. Each F_4_ family was derived via SSD, with OC/PC measured on bulk seeds. Plots were single-row (4.5 m × 0.5 m row spacing) without replication; border rows minimized edge effects, and standard agronomic practices ensured reliable phenotypic data.

### 4.2. Phenotype Data Collection and Analysis

Near infra-red (NIR) calibration models for OC/PC were built using 200 diverse soybean accessions (excluding 4404 F_4_ lines), with reference values from Soxhlet apparatus (Büchi Labortechnik AG, Flawil, Switzerland) for OC and Kjeldahl analyzer (FOSS Analytical, Hillerød, Denmark) for PC. Partial least squares (PLS) regression with full cross-validation was used, optimized by predicted sum of squares (PRESS). External validation (50 samples) showed: OC (root mean square error of calibration-RMSEC = 0.30%, root mean square error of prediction-RMSEP = 0.50%, coefficient of determination for thecalibration set-R^2^_cal = 0.99, coefficient of determination for the validation-R^2^_val = 0.97); and PC (RMSEC = 0.63%, RMSEP = 0.52%, R^2^_cal = 0.98, R^2^_val = 0.95). Spectral ranges (4100–4940, 5390–6690, 6900–7130, 7185–9000 cm^−1^) excluded water bands; standard normal variate (SNV) and detrending corrected scatter. Models were applied to F_4_ lines. OC/PC density maps (ggpubr R package, within R v3.6.0), descriptive statistics (psych “describe.by”), and Pearson’s r (psych “cor”) were analyzed in R (v3.6.0, https://www.r-project.org/, accessed 20 April 2020).

### 4.3. Genotyping Analysis

Genomic DNA was extracted via cetyltrimethylammonium bromide-CTAB method [43]. Genotyping used a 20K chip (MOLBREEDING Biotechnology Co., Ltd., Beijing, China), with 20,659 SNPs selected (missing rate ≤ 20%, heterozygosity ≤ 30%, allele frequency 5–95%; 50 kb sliding window, coding region preference). Of these, 19,954 SNPs were called; and filtering (missing rate > 10%, minor allele frequency-MAF < 0.05) retained 9942 high-quality SNPs (Table S6).

### 4.4. Phylogenetic Relationship and PCA

Phylogenetic analysis was performed by FastTree (v2.2, http://www.microbesonline.org/fasttree/, accessed 20 April 2020) [44], visualization ggtree (R package) [45] and PCA PLINK (v1.9, https://www.cog-genomics.org/plink/, accessed 20 April 2020) [46] were performed using 9942 SNPs to infer population genetic structure.

### 4.5. MTA Analysis

MTA was done by using mrMLM v3.6.0 package (https://cran.r-project.org/web/packages/mrMLM/, accessed 21 April 2020) [47] with six multi-locus methods. Critical *p*-values: 0.01 (except FASTmrEMMA, 0.005); LOD = 3.0 (*p <* 0.0002) for significant QTNs. The first six PCs (capturing genetic variation, Figure 1D) and kinship matrix controlled population structure. Consensus QTNs were detected by ≥2 methods, or single method (LOD ≥ 3.0, ≤50 kb from annotated genes). LD decay (≈450 kb) set 500 kb as the clustering threshold; redundant QTNs were merged, retaining the highest LOD markers, yielding 83 (OC) and 110 (PC) non-redundant QTNs.

### 4.6. Genomic Selection

GS models (rrBLUP, BayesA/B/C, BL, BRR) were implemented in R (v3.6.0) via BGLR [48] and rrBLUP [7]. Ten independent 10-fold cross-validations compared prediction accuracies, calculated as mean correlation of predicted/observed values per replicate.

## 5. Conclusions

In summary, this study presents a new strategy that integrating whole-genome marker association analysis with QTN-assisted GS to reduce genotyping costs as well as mitigates the negative genetic correlation between OC and PC in soybean. However, our findings are based on a single environment and a specific set of multi-parent populations derived from seven elite cultivars. For commercial applications of this strategy in the crop breeding, there is need for its validation across multiple environments and independent breeding populations. Nevertheless, our results provide a roadmap for future efforts to simultaneously improve negatively correlated traits in soybean and other crops.

## Data Availability

The original contributions presented in this study are included in the article. Further inquiries can be directed to the corresponding authors.

## Supplementary Materials

The following supporting information can be downloaded at https://www.mdpi.com/article/10.3390/plants15091296/s1, Figure S1. Schematic workflow diagram. Figure S2. QQ plot of the oil content and protein content. Figure S3. Distribution of QTN and SNP effect for genomics selection. Figure S4. Robustness analysis of GS models using full versus filtered QTN sets. Table S1. Six F_4_ populations derived from seven parents. Table S2. QTNs detected from MTA analysis. Table S3. Comparison of predictive accuracy between SNP and QTNS Markers. Table S4. Comparison of prediction accuracy between QTN set and filtered QTN. Table S5. Comparison of genomic prediction accuracy among three marker panels. Table S6. Summary of SNP quality control (QC) filtering.

## Abbreviations

The following abbreviations are used in this manuscript:

OC	Oil content
PC	Protein content
QTNs	Quantitative trait nucleotides
GS	Genomic selection
TP	Training populations
