# Supplementary material for: A Quantitative Trait Nucleotide-Based Genomic Selection Strategy for Seed Oil and Protein Content in Soybean
Source: Plants (Basel). 2026 Apr 22;15(9):1296. doi: 10.3390/plants15091296 (PMC13165166; doi:10.3390/plants15091296)
Supplement: Supplementary file 1 [file plants-15-01296-s001.zip › plants-4102520-supplementary.pdf]

Supplementary Materials

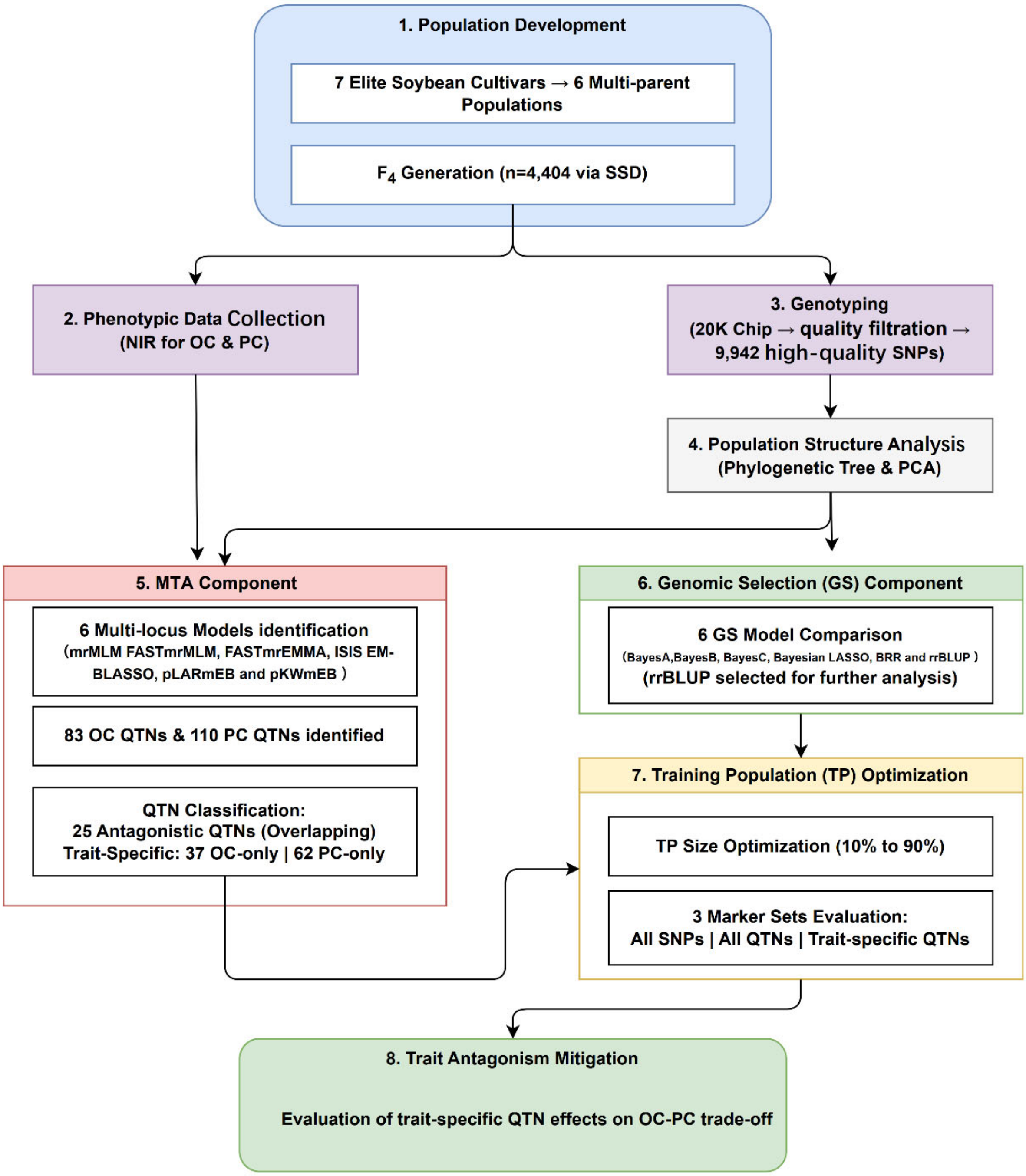

Figure S1. Schematic workflow diagram.

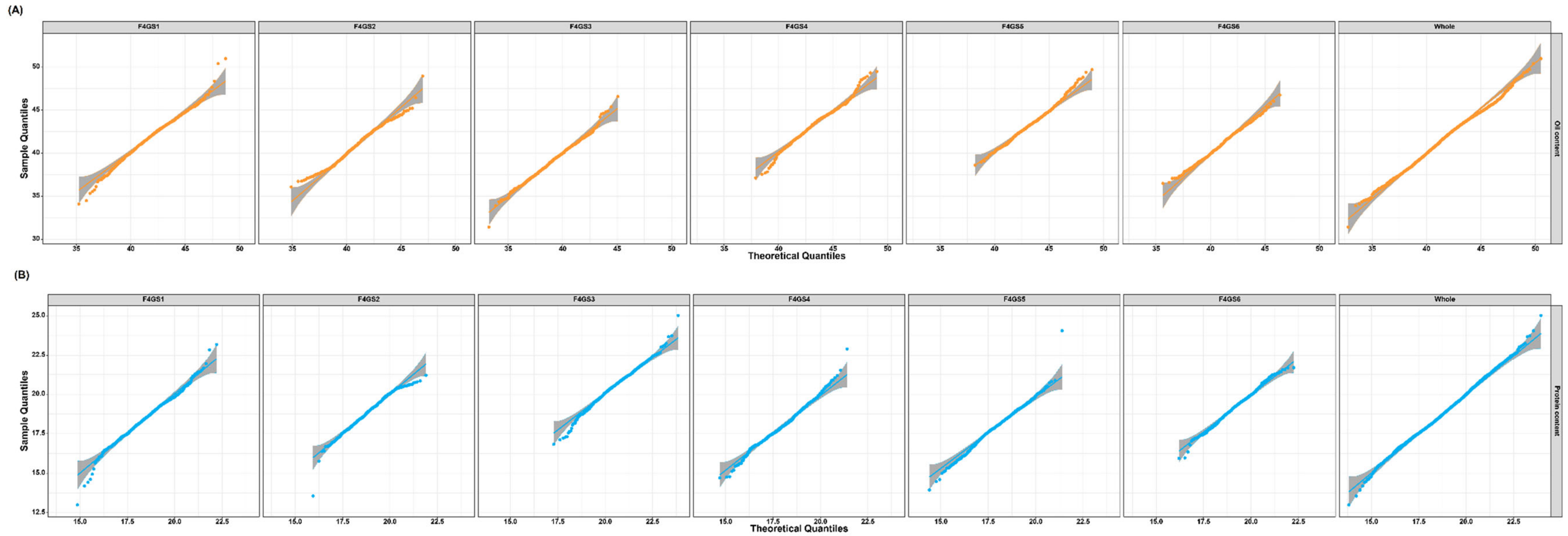

Figure S2. QQ plot of the oil content and protein content.

Notes:

1. Oil content(A) and Protein content(B).

2. The X-axis is the theoretical value under normal distribution; Y-axis is the actual value.

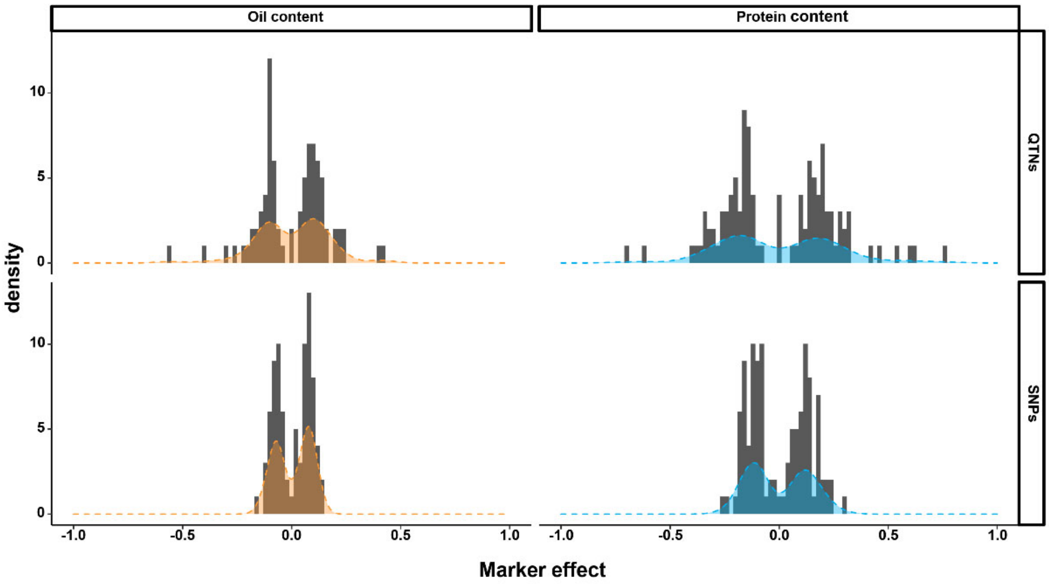

Figure S3. Distribution of QTN and SNP effect for genomics selection. The x-axis represents the estimated marker effects for oil content (OC) or protein content (PC). The y-axis shows the kernel density estimate.

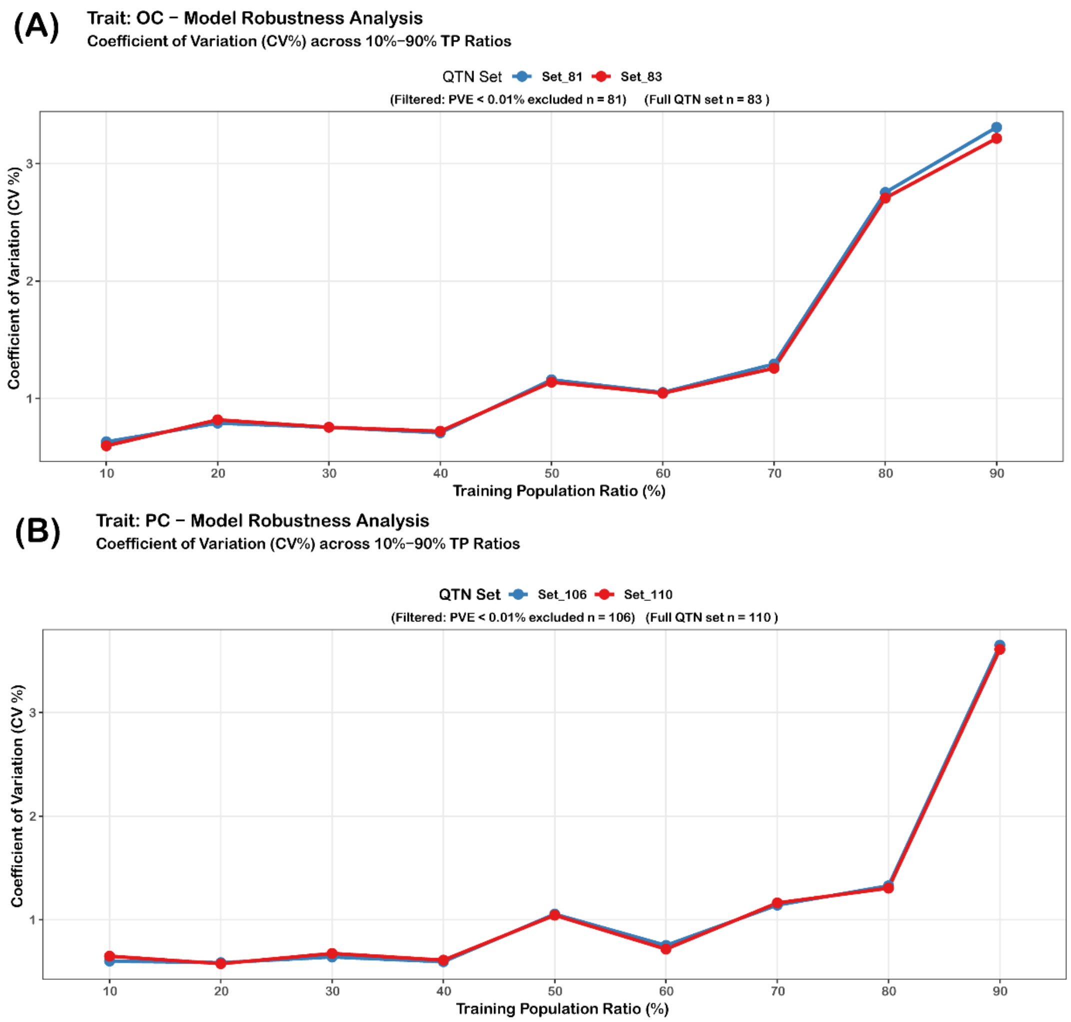

Figure S4. Robustness analysis of GS models using full versus filtered QTN sets.

Notes:

Oil content(A) and Protein content(B).

Table S1. Six F_4 populations derived from seven parents.

Population	Size	Male parent	Oil (%)	Protein (%)	Female parent	Oil (%)	Protein (%)	Correlation coefficient
F_4GS1	820	FNGS0225	21.60	40.95	FNGS0852	19.35	44.64	-0.7727451
F_4GS2	676	FNGS0225	21.60	40.95	FNGS0256	20.94	44.35	-0.7762585
F_4GS3	781	FNGS0217	22.01	40.72	FNGS0280	18.52	44.84	-0.8128338
F_4GS4	707	FNGS0217	22.01	40.72	FNGS0301	17.90	46.69	-0.8196748
F_4GS5	723	FNGS0239	19.67	43.46	FNGS0256	20.94	44.35	-0.7562378
F_4GS6	697	FNGS0239	19.67	43.46	FNGS0280	18.52	44.84	-0.7609687

Table S2. QTNs detected from MTA analysis.

Trait	Marker	Chromosome	Marker position (bp)	QTN effect mean	r^2 (%)	MAF	Overlapping
OC	1_1550268	1	1550268	-0.122175	0.6065	0.4022	Yes
OC	1_1656913	1	1656913	-0.1001	0.5854	0.3468	Yes
OC	1_52250882	1	52250882	-0.0827	0.264	0.2889	No
OC	1_54530855	1	54530855	0.23765	1.4223	0.1662	No
OC	2_1275126	2	1275126	0.1085	1.246	0.3883	No
OC	2_3200625	2	3200625	0.1281	0.7364	0.2699	Yes
OC	2_4425394	2	4425394	0.1501	0.9024	0.2242	No
OC	2_5184206	2	5184206	-0.0803	0.337	0.4212	No
OC	2_6300927	2	6300927	0.112	0.0969	0.3862	No
OC	2_46776729	2	46776729	0.0001	6.6589E-07	0.4749	No
OC	2_46906445	2	46906445	0.000034369	3.749E-08	0.2139	No
OC	2_47075851	2	47075851	0.0608	0.1192	0.2222	No
OC	3_3826007	3	3826007	0.0898	0.3627	0.24	No
OC	3_29819202	3	29819202	0.0301	0.0278	0.1542	Yes
OC	3_34059598	3	34059598	0.0748	0.1448	0.1688	Yes
OC	3_34108558	3	34108558	0.0712	0.1347	0.1713	Yes
OC	3_36421392	3	36421392	-0.1731	0.7528	0.1771	Yes
OC	3_36504983	3	36504983	-0.5611	2.1857	0.1802	No
OC	3_43061605	3	43061605	-0.103	1.0543	0.4073	No
OC	3_44975858	3	44975858	0.39702	1.6093	0.2144	No
OC	4_5839404	4	5839404	0.2324	0.3921	0.3717	Yes
OC	4_8600187	4	8600187	0.1456	0.419	0.1282	Yes
OC	4_8834618	4	8834618	0.15185	0.4871	0.1308	Yes
OC	4_47157109	4	47157109	-0.1068	0.4193	0.2047	No
OC	5_28002461	5	28002461	0.2173	0.8314	0.088	No
OC	5_33525340	5	33525340	0.1269	0.2828	0.1638	No
OC	5_41175611	5	41175611	0.0694	0.2679	0.4395	Yes
OC	6_4351486	6	4351486	0.143475	0.3472	0.302	Yes
OC	6_5766823	6	5766823	-0.3952	0.6422	0.097	Yes
OC	6_6078531	6	6078531	-0.113	0.1986	0.099	Yes
OC	6_13003203	6	13003203	-0.1307	0.6862	0.2947	Yes
OC	6_13672550	6	13672550	-0.091	0.2095	0.1599	Yes
OC	6_14058279	6	14058279	-0.0988	0.1996	0.13	Yes
OC	6_17626369	6	17626369	0.122033333	0.7131	0.3699	Yes
OC	6_19823381	6	19823381	-0.210233333	2.09	0.335	Yes
OC	6_42000621	6	42000621	-0.19986	0.1403	0.0707	No
OC	7_35977735	7	35977735	-0.092	0.3161	0.2676	No
OC	7_36951473	7	36951473	0.0706	0.1966	0.2971	No
OC	7_38653177	7	38653177	0.0648	0.1799	0.2461	No
OC	8_1798324	8	1798324	0.0951	0.4042	0.2716	No
OC	8_6659330	8	6659330	0.081	0.3192	0.4119	No

OC	8_14386629	8	14386629	0.0562	0.1524	0.4072	No
OC	8_44725861	8	44725861	-0.084	0.2438	0.2339	Yes
OC	8_46278339	8	46278339	-0.264225	0.5002	0.0735	Yes
OC	9_925073	9	925073	-0.0979	0.4643	0.3823	Yes
OC	9_2909053	9	2909053	0.0475	0.1149	0.3504	Yes
OC	9_5001601	9	5001601	0.198466667	0.4088	0.4453	Yes
OC	9_36783286	9	36783286	0.1001	0.7019	0.342	No
OC	9_41576646	9	41576646	-0.0725	0.1952	0.335	No
OC	9_42254662	9	42254662	-0.0931	0.4563	0.4263	No
OC	9_43775281	9	43775281	-0.141	0.6791	0.2346	No
OC	9_44322265	9	44322265	-0.303566667	1.3938	0.2016	No
OC	9_48480888	9	48480888	0.1442	0.4428	0.1374	Yes
OC	9_48808081	9	48808081	0.1402	0.4305	0.1411	Yes
OC	10_1707438	10	1707438	0.426225	0.3535	0.1849	Yes
OC	11_4411689	11	4411689	0.0459	0.102	0.417	No
OC	11_32115772	11	32115772	-0.1145	0.4299	0.1698	No
OC	12_34137308	12	34137308	0.0868	0.3981	0.4323	Yes
OC	12_34851455	12	34851455	-0.1518	1.095	0.3258	Yes
OC	12_36736475	12	36736475	0.10685	0.3206	0.3609	No
OC	12_36851478	12	36851478	0.0822	0.7346	0.3133	No
OC	12_39108395	12	39108395	0.1171	0.7466	0.4648	Yes
OC	12_39228628	12	39228628	0.0909	0.4283	0.4959	Yes
OC	14_46703357	14	46703357	-0.1896	0.8897	0.0756	Yes
OC	15_4541989	15	4541989	0.1074	0.608	0.4839	Yes
OC	15_6750342	15	6750342	-0.0647	0.221	0.4636	Yes
OC	15_8671170	15	8671170	-0.1527	1.0484	0.3575	Yes
OC	15_11213229	15	11213229	-0.0943	0.45	0.3449	Yes
OC	15_11356554	15	11356554	-0.0501	0.1315	0.4942	Yes
OC	15_16793506	15	16793506	0.203733333	0.5302	0.3291	No
OC	15_20567865	15	20567865	0.1287	0.539	0.2163	Yes
OC	15_21277170	15	21277170	0.1337	0.6781	0.2592	Yes
OC	16_1250592	16	1250592	-0.0483	0.0994	0.3154	No
OC	16_36854499	16	36854499	-0.0709	0.2382	0.2923	No
OC	17_9779938	17	9779938	0.0644	0.1866	0.3572	Yes
OC	17_11025426	17	11025426	-0.1133	0.3799	0.1924	No
OC	17_15250595	17	15250595	-0.0909	0.4072	0.3988	Yes
OC	17_36354958	17	36354958	-0.1082	0.9801	0.4571	No
OC	18_7080864	18	7080864	-0.1032	0.3779	0.3987	Yes
OC	18_54801747	18	54801747	-0.077	0.6549	0.4973	Yes
OC	18_55800924	18	55800924	-0.1393	0.6237	0.3888	Yes
OC	19_48127495	19	48127495	0.211	0.6789	0.0541	Yes
OC	20_1894103	20	1894103	0.09455	0.1555	0.1606	Yes
PC	1_1550268	1	1550268	0.125633333	0.2798	0.4022	Yes
PC	2_2986388	2	2986388	0.27205	0.4085	0.0802	Yes

PC	2_11075770	2	11075770	-0.1532	0.3512	0.0751	No
PC	2_15581360	2	15581360	-0.1949	0.3448	0.178	No
PC	3_29819202	3	29819202	-0.1581	0.251	0.1542	Yes
PC	3_34077256	3	34077256	-0.1707	0.253	0.1711	Yes
PC	3_36421392	3	36421392	0.194725	0.7371	0.1772	Yes
PC	3_37706557	3	37706557	0.1591	0.3152	0.2842	No
PC	4_4000319	4	4000319	0.2336	0.0778	0.0819	No
PC	4_5839404	4	5839404	-0.25455	1.8036	0.3719	Yes
PC	4_8600187	4	8600187	-0.3236	0.3507	0.1282	Yes
PC	4_8834618	4	8834618	-0.252866667	0.549	0.1308	Yes
PC	4_41995829	4	41995829	-0.10585	0.6433	0.3845	No
PC	5_2612793	5	2612793	0.53442	2.5253	0.3067	No
PC	5_2667653	5	2667653	-0.2977	1.2213	0.299	No
PC	5_3614833	5	3614833	0.203866667	0.1974	0.2445	No
PC	5_31775728	5	31775728	0.2822	0.3296	0.0754	No
PC	5_41586392	5	41586392	-0.0866	0.1238	0.3412	Yes
PC	6_432211	6	432211	0.3269	0.7978	0.1013	No
PC	6_1052684	6	1052684	-0.26665	2.1718	0.4575	No
PC	6_1129202	6	1129202	0.2	0.7851	0.2776	No
PC	6_1203733	6	1203733	-0.23945	0.8531	0.1746	No
PC	6_4334059	6	4334059	-0.1378	0.4401	0.2991	Yes
PC	6_5766823	6	5766823	0.4593	1.0443	0.097	Yes
PC	6_6078531	6	6078531	0.411633333	0.9173	0.0989	Yes
PC	6_6321130	6	6321130	0.7656	0.823	0.101	Yes
PC	6_11878270	6	11878270	-0.2492	0.4471	0.1364	No
PC	6_13003203	6	13003203	0.303166667	1.2383	0.2949	Yes
PC	6_13412419	6	13412419	0.2884	0.5163	0.2623	Yes
PC	6_13672550	6	13672550	0.1949	0.315	0.1599	Yes
PC	6_17582094	6	17582094	-0.3818	2.4514	0.3694	Yes
PC	6_17626369	6	17626369	-0.69778	5.0288	0.3699	Yes
PC	6_19275883	6	19275883	0.1306	0.2922	0.4046	Yes
PC	6_46153054	6	46153054	0.216	0.2165	0.0884	No
PC	6_48242356	6	48242356	0.1057	0.1106	0.2083	No
PC	7_16889053	7	16889053	0.1736	0.3646	0.1994	No
PC	8_9776456	8	9776456	-0.1205	0.147	0.2003	No
PC	8_10675535	8	10675535	-0.1135	0.1951	0.3748	No
PC	8_15532658	8	15532658	-0.1172	0.1835	0.3099	No
PC	8_22426734	8	22426734	-0.2149	0.4228	0.1855	No
PC	8_22750629	8	22750629	0.2469	0.7461	0.2768	No
PC	8_42281579	8	42281579	0.0941	0.092	0.218	No
PC	8_42977728	8	42977728	0.0937	0.0791	0.178	No
PC	8_44725861	8	44725861	0.00008487	8.1476E-08	0.2339	Yes
PC	8_46278339	8	46278339	0.232666667	0.4073	0.0735	Yes
PC	9_925073	9	925073	0.1914	0.5817	0.3823	Yes

PC	9_1128170	9	1128170	0.1604	0.4076	0.3803	No
PC	9_3165826	9	3165826	0.1337	0.2884	0.4276	Yes
PC	9_5001601	9	5001601	-0.1311	0.2961	0.4453	Yes
PC	9_5418798	9	5418798	0.1371	0.3247	0.4427	Yes
PC	9_40078265	9	40078265	-0.613	5.7832	0.2514	No
PC	9_40521623	9	40521623	0.2513	0.9563	0.1837	No
PC	9_47769078	9	47769078	-0.2076	0.6157	0.1398	Yes
PC	9_49079942	9	49079942	-0.1975	0.2898	0.1449	Yes
PC	9_49162387	9	49162387	-0.2166	0.3507	0.1466	Yes
PC	10_789952	10	789952	-0.1664	0.4085	0.3532	Yes
PC	10_1707438	10	1707438	-0.1952	0.3626	0.1849	Yes
PC	10_3373357	10	3373357	-0.154	0.4106	0.4545	No
PC	10_39927065	10	39927065	-0.334	2.594	0.0734	No
PC	12_34137308	12	34137308	-0.1365	0.8816	0.4324	Yes
PC	12_34756687	12	34756687	-0.1487	0.3394	0.3748	Yes
PC	12_34851455	12	34851455	0.320975	1.6903	0.3256	Yes
PC	12_34957065	12	34957065	0.63	1.4023	0.322	Yes
PC	12_39228628	12	39228628	-0.152	0.3925	0.4959	Yes
PC	13_13308150	13	13308150	0.1052	0.403	0.2543	No
PC	13_18011254	13	18011254	-0.000065381	7.0449E-08	0.3137	No
PC	13_18400146	13	18400146	0.0807	0.0907	0.2444	No
PC	13_28577222	13	28577222	0.1332	0.1903	0.1687	No
PC	13_33375272	13	33375272	0.183033333	0.3416	0.2062	No
PC	13_34276074	13	34276074	-0.2351	0.9363	0.3924	No
PC	13_37740952	13	37740952	0.1731	0.3663	0.1813	No
PC	13_44000635	13	44000635	-0.1384	0.3119	0.4305	No
PC	13_44112040	13	44112040	0.1861	1.0421	0.3877	No
PC	14_6428414	14	6428414	0.12715	0.2032	0.3442	No
PC	14_15620568	14	15620568	-0.1471	1.2293	0.2958	No
PC	14_16944874	14	16944874	-0.1529	0.4021	0.4872	No
PC	14_30825490	14	30825490	-0.1323	0.2842	0.312	No
PC	14_46775040	14	46775040	-0.22845	0.9265	0.2569	Yes
PC	15_1856253	15	1856253	0.2921	0.3237	0.0635	No
PC	15_1926315	15	1926315	2.101	4.2654	0.0627	No
PC	15_2030959	15	2030959	0.606	1.3937	0.0626	No
PC	15_8671170	15	8671170	0.1571	2.2728	0.3575	Yes
PC	15_11356554	15	11356554	0.1307	0.2929	0.4942	Yes
PC	15_11441207	15	11441207	-0.367233333	2.1167	0.2162	Yes
PC	15_21277170	15	21277170	-0.3343	1.7533	0.2592	Yes
PC	15_24039112	15	24039112	-0.3032	1.2878	0.2438	No
PC	15_43522418	15	43522418	0.1525	0.2546	0.3427	No
PC	16_4075059	16	4075059	-0.277233333	1.6148	0.3526	No
PC	16_18832728	16	18832728	-0.1267	0.2363	0.2687	No
PC	16_27803269	16	27803269	-0.2039	0.7163	0.4185	No

PC	16_29035313	16	29035313	-0.1831	0.2708	0.1097	No
PC	16_31675884	16	31675884	-0.1344	1.2557	0.261	No
PC	16_32516100	16	32516100	0.207	0.4041	0.25	No
PC	16_33682382	16	33682382	0.0526	0.0467	0.3602	No
PC	17_1680401	17	1680401	0.219	0.3884	0.0847	No
PC	17_9838390	17	9838390	-0.155733333	0.2979	0.3565	Yes
PC	17_15250595	17	15250595	0.1406	0.3389	0.3988	Yes
PC	18_7758619	18	7758619	0.158	1.8847	0.4435	Yes
PC	18_8228210	18	8228210	-0.1521	0.389	0.4528	No
PC	18_53504757	18	53504757	-0.1877	0.417	0.2582	No
PC	18_53624805	18	53624805	-0.3121	1.7142	0.4228	No
PC	18_54853314	18	54853314	-0.3977	1.1225	0.1503	Yes
PC	18_54937497	18	54937497	0.21625	0.7585	0.3902	Yes
PC	18_55800924	18	55800924	0.32475	0.7299	0.3886	Yes
PC	18_57743617	18	57743617	-0.1604	0.1784	0.1333	No
PC	19_9281354	19	9281354	-0.0047	0.0004	0.4448	No
PC	19_35100145	19	35100145	-0.000029506	1.2874E-08	0.3378	No
PC	19_40177289	19	40177289	0.1986	0.6723	0.4251	No
PC	20_1750542	20	1750542	-0.21265	0.5696	0.3363	Yes
PC	20_1894103	20	1894103	-0.33005	0.8894	0.1607	Yes

Notes:
1. **QTN effect mean**: The average additive genetic effect of the marker on oil content (OC). A positive value indicates that the minor allele increases OC, while a negative value indicates a decrease.
2. **r^2 (%)**: Percentage of phenotypic variance explained (PVE) by each individual QTN.
Marker Position: Physical coordinates of the SNPs based on the soybean reference genome (e.g., Wm82.a2.v1).
3. **MAF**: Minor Allele Frequency, representing the frequency of the less common allele in the natural population
4. **Overlapping**: Indicates whether the QTN region coincides with previously reported loci or QTNs identified for related traits. "Yes" signifies a potential pleiotropic or linked locus, while "No" suggests a trait-specific QTN.

Table S3. Comparison of predictive accuracy between SNP and QTNS Markers

Marker type	Trait	Comparison	*P-value*	Adjusted *P-value*	Significant
SNPs	OC	BayesA vs BayesB	0.312014961	1	No
SNPs	OC	BayesA vs BayesC	0.948609723	1	No
SNPs	OC	BayesA vs BL	0.891639153	1	No
SNPs	OC	BayesA vs BRR	0.034826795	0.522401931	No
SNPs	OC	BayesA vs rrBLUP	0.109757284	1	No
SNPs	OC	BayesB vs BayesC	0.219016325	1	No
SNPs	OC	BayesB vs BL	0.189141072	1	No
SNPs	OC	BayesB vs BRR	0.862176709	1	No

SNPs	OC	BayesB vs rrBLUP	0.734890907	1	No
SNPs	OC	BayesC vs BL	0.782836001	1	No
SNPs	OC	BayesC vs BRR	0.111556135	1	No
SNPs	OC	BayesC vs rrBLUP	0.199535717	1	No
SNPs	OC	BL vs BRR	0.115051799	1	No
SNPs	OC	BL vs rrBLUP	0.195605629	1	No
SNPs	OC	BRR vs rrBLUP	0.73972373	1	No
SNPs	PC	BayesA vs BayesB	0.503599826	1	No
SNPs	PC	BayesA vs BayesC	0.937181813	1	No
SNPs	PC	BayesA vs BL	0.987187081	1	No
SNPs	PC	BayesA vs BRR	0.801579147	1	No
SNPs	PC	BayesA vs rrBLUP	0.436372978	1	No
SNPs	PC	BayesB vs BayesC	0.607784187	1	No
SNPs	PC	BayesB vs BL	0.367800977	1	No
SNPs	PC	BayesB vs BRR	0.506978048	1	No
SNPs	PC	BayesB vs rrBLUP	0.920607336	1	No
SNPs	PC	BayesC vs BL	0.945834211	1	No
SNPs	PC	BayesC vs BRR	0.92455323	1	No
SNPs	PC	BayesC vs rrBLUP	0.663150851	1	No
SNPs	PC	BL vs BRR	0.877330648	1	No
SNPs	PC	BL vs rrBLUP	0.56472386	1	No
SNPs	PC	BRR vs rrBLUP	0.519570181	1	No
QTNs	OC	BayesA vs BayesB	0.5142724	1	No
QTNs	OC	BayesA vs BayesC	0.173946985	1	No
QTNs	OC	BayesA vs BL	0.813647175	1	No
QTNs	OC	BayesA vs BRR	0.424251117	1	No
QTNs	OC	BayesA vs rrBLUP	0.404945555	1	No
QTNs	OC	BayesB vs BayesC	0.142864337	1	No
QTNs	OC	BayesB vs BL	0.700435769	1	No
QTNs	OC	BayesB vs BRR	0.965095598	1	No
QTNs	OC	BayesB vs rrBLUP	0.825391867	1	No
QTNs	OC	BayesC vs BL	0.261962912	1	No
QTNs	OC	BayesC vs BRR	0.281596537	1	No
QTNs	OC	BayesC vs rrBLUP	0.20319284	1	No
QTNs	OC	BL vs BRR	0.634089737	1	No
QTNs	OC	BL vs rrBLUP	0.460468928	1	No
QTNs	OC	BRR vs rrBLUP	0.738765222	1	No
QTNs	PC	BayesA vs BayesB	0.119324641	1	No
QTNs	PC	BayesA vs BayesC	0.189353523	1	No
QTNs	PC	BayesA vs BL	0.921539751	1	No
QTNs	PC	BayesA vs BRR	0.592819546	1	No
QTNs	PC	BayesA vs rrBLUP	0.936633713	1	No
QTNs	PC	BayesB vs BayesC	0.896101351	1	No
QTNs	PC	BayesB vs BL	0.167875035	1	No

QTNs	PC	BayesB vs BRR	0.343707176	1	No
QTNs	PC	BayesB vs rrBLUP	0.082276327	1	No
QTNs	PC	BayesC vs BL	0.137722857	1	No
QTNs	PC	BayesC vs BRR	0.527437199	1	No
QTNs	PC	BayesC vs rrBLUP	0.189316376	1	No
QTNs	PC	BL vs BRR	0.427372973	1	No
QTNs	PC	BL vs rrBLUP	0.966448781	1	No
QTNs	PC	BRR vs rrBLUP	0.450714788	1	No

Notes:

1. **Model Abbreviations**: BayesA, BayesB, and BayesC: Bayesian regression models with different prior distributions for marker effects; BL: Bayesian LASSO; BRR: Bayesian Ridge Regression; rrBLUP: Ridge Regression Best Linear Unbiased Prediction.

2. ***P-value***: Calculated using a paired t-test comparing the prediction accuracies (r) between two models across all cross-validation folds.

3. **Adjusted *P-value***: P-values were adjusted for multiple comparisons using the Bonferroni correction. Adjusted values exceeding 1.0 were truncated to 1.

4. **Significant**: "No" indicates that the difference in prediction performance between the two models is not statistically significant at the 0.05 level ($P > 0.05$) after multiple testing correction.

Table S4 Comparison of prediction accuracy between QTN set and filtered QTN.

**Trait**	**TP Ratio (%)**	**Mean (Filtered: PVE < 0.01% excluded)**	**Mean (Full QTN set)**	**Diff**	***P-value***	**Sig**
OC	10	0.728911428	0.728615308	-0.00029612	0.902557205	Not significant
OC	20	0.728352215	0.732004814	0.003652599	0.184283024	Not significant
OC	30	0.728330114	0.730726021	0.002395906	0.399894874	Not significant
OC	40	0.731104547	0.731666006	0.000561458	0.850112111	Not significant
OC	50	0.731279649	0.734470985	0.003191336	0.181115388	Not significant
OC	60	0.732204329	0.72942845	-0.002775879	0.293520752	Not significant
OC	70	0.729361676	0.732299998	0.002938321	0.307900415	Not significant
OC	80	0.729760951	0.732607156	0.002846205	0.30296761	Not significant
OC	90	0.728193305	0.731097726	0.002904421	0.249324277	Not significant
PC	10	0.731897507	0.728497268	-0.003400238	0.086374202	Not significant
PC	20	0.725207447	0.732226826	0.007019379	0.016690821	Not significant
PC	30	0.731508929	0.731182981	-0.000325947	0.888968357	Not significant
PC	40	0.734337799	0.72976255	-0.004575248	0.088998037	Not significant
PC	50	0.730805079	0.729327109	-0.001477971	0.588083994	Not significant
PC	60	0.730729854	0.72882137	-0.001908483	0.420383266	Not significant
PC	70	0.729832028	0.728291571	-0.001540457	0.496144375	Not significant
PC	80	0.731050103	0.732524794	0.001474691	0.549943957	Not significant
PC	90	0.729433848	0.728586322	-0.000847526	0.736590996	Not significant

Notes:

1. TP Ratio (%): Proportion of the total population used as the training set.
2. Filtered Set (PVE < 0.01% excluded): QTN set where markers explaining less than 0.01% of the phenotypic variance were removed.
3. Original Set: The complete set of identified QTNs used in the initial genomic selection model.
4. *P-value*: Calculated using a paired

5. t-test comparing the prediction accuracy (r) of the two sets across multiple iterations.
6. Significance (Sig): "ns" indicates not significant at the 0.01 level *($P>0.01$)*

Table S5. Comparison of genomic prediction accuracy among three marker panels.

Trait	Marker Panel	TP Size (%)	Mean PA	Mean PA	Significance Letter
OC	SNPs	10	0.715879	0.687411	a
OC	Special QTNs	10	0.693171	0.665529	b
OC	QTNs	10	0.746958	0.721445	c
OC	SNPs	20	0.727921	0.700071	a
OC	Special QTNs	20	0.70511	0.679568	b
OC	QTNs	20	0.75686	0.732455	c
OC	SNPs	30	0.739687	0.710753	a
OC	Special QTNs	30	0.713849	0.684174	b
OC	QTNs	30	0.76938	0.739514	c
OC	SNPs	40	0.748745	0.718849	a
OC	Special QTNs	40	0.718522	0.687626	b
OC	QTNs	40	0.773175	0.743195	c
OC	SNPs	50	0.74901	0.723734	a
OC	Special QTNs	50	0.720828	0.687444	b
OC	QTNs	50	0.781641	0.74588	c
OC	SNPs	60	0.76232	0.73171	a
OC	Special QTNs	60	0.718236	0.686842	b
OC	QTNs	60	0.770492	0.742141	c
OC	SNPs	70	0.764251	0.733067	a
OC	Special QTNs	70	0.722021	0.68893	b
OC	QTNs	70	0.786579	0.751017	c
OC	SNPs	80	0.77715	0.739804	a
OC	Special QTNs	80	0.747008	0.684185	b
OC	QTNs	80	0.787182	0.747376	a
OC	SNPs	90	0.789651	0.74126	a
OC	Special QTNs	90	0.742592	0.703564	b
OC	QTNs	90	0.797055	0.761289	c
PC	SNPs	10	0.724658	0.698448	a
PC	Special QTNs	10	0.719978	0.686312	b
PC	QTNs	10	0.772261	0.745509	c
PC	SNPs	20	0.75523	0.723369	a
PC	Special QTNs	20	0.731317	0.705205	b
PC	QTNs	20	0.784541	0.759003	c
PC	SNPs	30	0.763335	0.735578	a
PC	Special QTNs	30	0.739543	0.708343	b
PC	QTNs	30	0.792221	0.765433	c
PC	SNPs	40	0.768307	0.74382	a
PC	Special QTNs	40	0.735443	0.711893	b
PC	QTNs	40	0.792362	0.766935	c
PC	SNPs	50	0.77235	0.7438	a
PC	Special QTNs	50	0.744034	0.712928	b

PC	QTNs	50	0.800028	0.76531	c
PC	SNPs	60	0.784554	0.752572	a
PC	Special QTNs	60	0.743362	0.717165	b
PC	QTNs	60	0.804197	0.769467	c
PC	SNPs	70	0.781138	0.750343	a
PC	Special QTNs	70	0.760698	0.719034	b
PC	QTNs	70	0.806412	0.774993	c
PC	SNPs	80	0.797426	0.756005	a
PC	Special QTNs	80	0.757765	0.717295	b
PC	QTNs	80	0.81222	0.778153	c
PC	SNPs	90	0.81302	0.761898	a
PC	Special QTNs	90	0.763538	0.714447	b
PC	QTNs	90	0.828364	0.772224	a

Note:

1. **Marker Panels**: SNPs (GSall) refers to the genomic selection model using all 9,942 genome-wide markers; QTNs (GSQTN) includes all significant loci detected via multi-locus transition analysis (83 for OC, 110 for PC); Special QTNs represents a refined subset of markers specifically associated with the target trait (37 for OC, 62 for PC).

2. **Mean PA**: The average Pearson correlation coefficient between predicted and observed phenotypic values across multiple cross-validation folds.

3. **Max PA**: The highest prediction accuracy observed within the iterations for each training population (TP) size.

4. **Significance**: Different lowercase letters (a, b, c) within the same Trait and TP Size indicate significant differences among marker panels based on Tukey's HSD test ($p < 0.05$).

Table S6. Summary of SNP quality control (QC) filtering.

**Filtering step**	**Criterion**	**Markers removed**	**Markers remaining**
Initial	–	–	20,659
Genotyping success	–	705	19,954
Step 1	Missing rate > 10%	1358	18,596
Step 2	MAF < 0.05	8654	**9942**

Notes:

1. **Initial**: The total number of raw Single Nucleotide Polymorphism (SNP) markers obtained from the initial genotyping platform.

2. Genotyping success: Markers excluded due to poor technical performance, such as low call rates.

3. Missing rate: A threshold of > 10% indicates that markers with data missing in more than 10% of the population were removed.

4. MAF (Minor Allele Frequency): The frequency of the least common allele at a given locus. SNPs with MAF < 0.05 were excluded to minimize spurious associations and focus on common genetic variants.

5. Markers remaining: The final set of high-quality SNPs used for downstream analysis.
